# Beyond systemic lupus erythematosus: a systematic review on the use of belimumab in other connective tissue diseases and vasculitides

**DOI:** 10.1007/s10067-025-07847-5

**Published:** 2025-12-04

**Authors:** Filippo Santoro, Martina Orlandi, Giulia Cassone, Maria Grazia Malandra, Caterina Vacchi, Ottavio Secchi, Dilia Giuggioli

**Affiliations:** 1 Rheumatology Unit, Department of Medical and Surgical Sciences for Children and Adults, University of Modena and Reggio Emilia, Modena, Italy; 2 Rheumatology Unit, Azienda Ospedaliero-Universitaria Policlinico Di Modena, Modena, Italy; 3 School of Medicine, University Hospital of Modena and Reggio Emilia, Via del Pozzo 71, Modena, 41125 Italy

**Keywords:** Belimumab, BlySS, Connective tissue disease, Vasculitis

## Abstract

**Introduction:**

Belimumab is a monoclonal antibody with proven efficacy in systemic lupus erythematosus. However, its role in other connective tissue diseases or vasculitides is still not clear.

**Methods:**

A systematic literature review (SLR) of original research article up to March 22 2025 was performed according to the Preferred Reporting Items for Systematic Reviews and Meta-Analyses (PRISMA) guidelines. Studies were selected according to the Patients Exposure Outcome (PEO) framework and evaluated by quality assessment of diagnostic accuracy studies (QUADAS).

**Results:**

Thirty-seven articles were identified as relevant and assessed for eligibility. After full evaluation, 15 articles were selected to be included in the review.

**Conclusion:**

The SLR showed that data on the use of belimumab in rheumatic conditions, other than SLE, are still scarce. However, belimumab may have therapeutic potential in specific subsets of patients with rheumatic diseases characterized by aberrant B cell activation. Future research should focus on large, randomized phase 3 trials with extended follow-up to better define the role of belimumab in rheumatic diseases beyond SLE, particularly in combination with standard-of-care therapies.

**Supplementary information:**

The online version contains supplementary material available at 10.1007/s10067-025-07847-5.

## Introduction

Belimumab is a human IgG1λ monoclonal antibody that inhibits B cell activating factor (BAFF), modulating B cell function through mechanisms distinct from those of anti-CD20 agents. In SLE, both belimumab and the newer biological agent, anifrolumab, have completely changed the approach to disease treatment, reducing damage accrual and achieving higher rates of remission [1–5]. However, in other connective tissue diseases (CTDs) or vasculitides, we are still lacking targeted therapies to significantly reduce disease burden and damage. Furthermore, B cell depleting therapies, that are extremely effective in several autoimmune disorders [6–9], are associated with significant adverse events [10, 11]. Belimumab works by regulating B cells rather than peripheral destruction [12], allowing a lower infectious risk. Considering an alternative way to regulate B cells function may then be useful to provide an effective treatment even in frail patients where rituximab’s risks outweigh its benefits.

Considering its efficacy in SLE, it makes sense to evaluate the role for this monoclonal antibody in the treatment of other rheumatic conditions. In this systematic review, we aim to summarize the current evidence on the use of belimumab in rheumatological conditions beyond SLE.

## Methods

### Methodology and quality assessment

The study protocol was developed according to the Preferred Reporting Items for Systematic Reviews and Meta-Analyses (PRISMA) guidelines. Studies were selected according to the Patients Exposure Outcome (PEO) framework for eligibility of research question outlined in the supplementary material (Supplementary Table 1). Quality Assessment of Diagnostic Accuracy Studies (QUADAS) for articles included in the systematic review is summarized in Supplementary Table 2.

### Literature search strategy

PubMed, EMBASE, Cochrane, and ClinicalTrials. gov databases were employed for retrieving relevant publications. Each database was searched from the database inception date up until 21 st of March 2025.

The search query used for PubMed was the following: (Belimumab OR Blys) AND (vasculitis OR Sjogren OR myositis OR dermatomyositis OR Antisynthetase syndrome [Supplementary Concept] OR scleroderma OR sclerosis OR cryoglobulinemia).

For Embase, we amended the search query as follows: (‘belimumab’/exp OR belimumab OR blys) AND (‘vasculitis’/exp OR vasculitis OR sjogren OR ‘myositis’/exp OR myositis OR ‘dermatomyositis’/exp OR dermatomyositis OR ‘antisynthetase syndrome’/exp OR ‘antisynthetase syndrome’ OR (antisynthetase AND (‘syndrome’/exp OR syndrome) OR ‘scleroderma’/exp OR scleroderma OR ‘sclerosis’/exp OR sclerosis OR cryoglobulinemia). Finally, for Clinicaltrial.gov and Cochrane, the search query was: (Belimumab OR Blys) AND (vasculitis OR Sjogren OR myositis OR dermatomyositis OR Antisynthetase syndrome OR scleroderma OR sclerosis OR cryoglobulinemia).

### Eligibility criteria

**Fig. 1:**
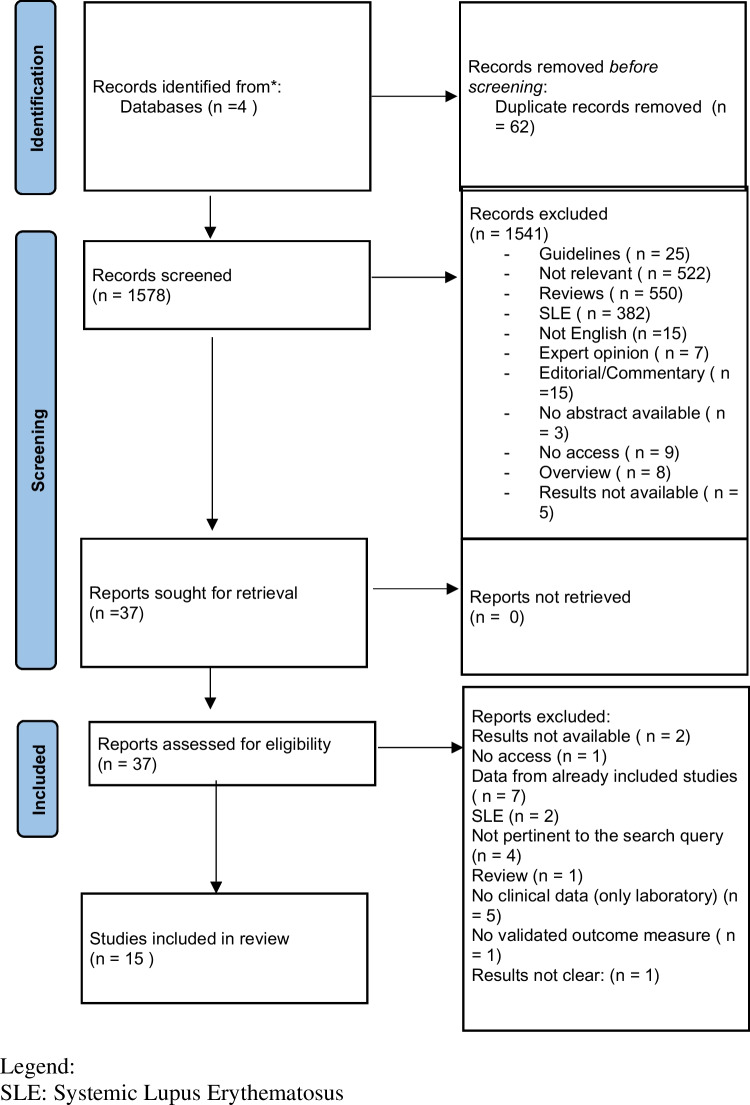
PRISMA flowchart. *SLE* systemic lupus erythematosus

Study inclusion criteria comprised peer-reviewed publication with population-based studies (Phase 1, phase 2 or phase 3 trials, and observational studies) reporting the use of Belimumab in CTDs and vasculitides. Case reports and case series were included due to the low prevalence of some diseases and the non-approved indication of the drug in said diseases. Congress abstracts, without a corresponding full text publication, were included provided that detailed data were reported. We only considered articles in English. A detailed flow chart describing the study inclusion and exclusion process is available in Fig. 1.

### Data extraction

All articles were identified by searching the databases and were imported to Mendeley for screening. After deduplication, two screening rounds were performed, as described in Fig. 1. In the first round, two reviewers (F.S. and G.C.) evaluated, in duplicate, titles and abstracts in terms of relevance for the review. In the second round, full texts of the articles included during the first round were retrieved and re-assessed for eligibility. Possible discordance during study selections were discussed with a third reviewer (M.O.) to reach a consensus. Only articles published in English and addressing the use of Belimumab in patients with systemic sclerosis (SSc), Sjogren’s syndrome (SS), inflammatory myositis, systemic vasculitis, and cryoglobulinemia were included.

Data were extracted and included in electronic database. The extracted contents were as follows: name of the first author, publication year, location of the studies, study design; study duration (months), availability and duration of follow up, total number of included patients in the study, number of people in the control population (if present), inclusion criteria, endpoints of the studies, outcomes, safety profile.

## Results

The initial search retrieved 199 publications from PubMed, 1336 from Embase, 76 from Cochrane, and 29 from Clinicaltrial.gov, for a total of 1640 articles.

After removal of duplicates, 1578 papers were uploaded into Mendeley. The first-stage screening by reviewing titles and abstracts excluded 1541 publications. Thirty-seven articles were identified as relevant and assessed for eligibility. Following the second-stage screening, 22 articles were excluded; reasons for exclusion are reported in Table 1. To capture other potentially relevant articles, we also evaluated the full list of references from other reviews and no articles were considered pertinent to our SLR. Finally, 15 articles were selected to be included in the review.

**Table 1:** Safety outcomes of the studies included

Author, year	Type of treatment	Total number of patients	Number of AEs/SAEs	AEs/SAEs in control group (if available)	Number control group (if available)
Yu, 2013 [25]	BEL + SOC	13 (6 DM and 7 JDM)	None		
Izuka, 2021 [22]	BEL + RTX	1 (SS associated cryoglobulinemic vasculitis)	None		
Chevalier, 2020 [20]	BEL + RTX	3 (SS cryoglobulinemic vasculitis)	None		
Jayne, 2019 [16]	BEL + AZA	54 (SSc patients)	49 AEs; 18 SAEs; 4 infectious complications	43 AEs; 16 SAEs; 4 infectious complications (no statistical difference)	52
Mariette, 2022 [15]	PLB (13 pts), BEL (24 pts), RTX (25 pts) BEL + RTX (24 pts)	86 (SS patients)	24 AEs (19 infections) and 3 SAEs in BEL + RTX; 23 AEs (21 infections), 2 SAEs in BEL; 24 AEs (18 infections), 7 SAEs in the RTX group	13 AEs (11 infections); no SAEs	13
Beketova, 2016 [17]	BEL (previous RTX and CYC)	1 (AAV)	None		
Belbezier, 2022 [27]	BEL	2 (SS)	None		
Marder, 2024 [26]	BEL + SOC (cDMARD and/or steroid)	10 (PM/DM)	17 AEs (2 infections), 1 SAE	15 AEs (5 infections), no SAEs	
de Vita, 2014 [19]	BEL + RTX	1 (SS)	None		
Mwangi, 2021 [24]	BEL + AZA	1 (overlap SSc/SLE)	None		
Gordon, 2019 [23]	BEL + MMF	9 (SSc)	None		
Saadoun, 2021 [21]	BEL + RTX	4 (cryoglobulinemic vasculitis)	1 pt: osteitis; 1 pt: recurrent UTI		
de Vita, 2015 [14]	BEL + SOC	28 (SS)	37 AEs, 1 SAE (pneumococcal meningitis)		
Mariette, 2013 [13]	BEL + SOC	28 (SS)			
Pouchelon, 2022 [18]	4 pts BEL; 7 pts BEL + RTX	11 (cryoglobulinemic vasculitis)	4 severe infections in the BEL + RTX; none in the BEL group		

*AAV* ANCA associated vasculitis

*AE* adverse event

*BEL* belimumab

*cDMARD* conventional disease modifying anti-rheumatic drug

*CYC* cyclophosphamide

*DM* dermatomyositis

*PLB* placebo

*PM* polymyositis

*RTX* rituximab

*SAE* severe adverse event

*SLE* systemic lupus erythematous

*SOC* standard of care

*SS* Sjogren’s syndrome

*SSc* systemic sclerosis

Figure 1 displays the flow-chart with details on the included and excluded papers.

### Safety

There was no significant association between belimumab use and adverse events. In the BELISS-2 trial [13, 14], the incidence of adverse events (AEs) was higher than expected; however, most AEs (mostly headaches and upper respiratory or gastrointestinal infections) were not severe.

Jayne et al. also reported a higher number of AEs in the treated group compared to the placebo arm; however, the risk of AEs—especially infectious ones—seemed to be associated more with the induction regimen. Regarding neoplastic risks, four patients in the belimumab group developed cancer; however, all patients were previously treated with cyclophosphamide.

The study by Mariette et al. [15] also demonstrated that belimumab was safe, as placebo and treatment arms did not differ. Furthermore, there was no difference among treatment arms, especially between the belimumab arm vs. the belimumab + rituximab arm. Safety outcomes of the studies included in the review are summarized in Table 1.

### Efficacy

Efficacy outcomes of the studies included in the review are summarized in Table 2.

**Table 2:** Characteristics of the studies included in the present SLR

Author, Year	Population	Type of study	Intervention	Inclusion criteria	Controls	Endpoints	Outcome	Other immunosuppressive treatment	Duration
Studies focusing on Sjogren’s syndrome									
Mariette, 2013 [13]	28 pts	phase II	Patients received belimumab, 10 mg/kg, at week (W) 0, 2, and 4 and then every 4 weeks to W24. Responders at W28 continued with belimumab monthly through W48	Diagnosis of SS (ACR/EULAR cirteria;) at least one: systemic complications or persistent salivary gland enlargement; early disease (≤ 5 years from the beginning of symptoms); presence of at least one biomarker of B cell activation		Primary: > 30% improvement, evaluated on a (VAS) in fatigue, musculoskeletal pain, systemic activity score; ≥ 25% reduction in serum levels of B cell activation biomarkers Secondary: change in the following items: each of the five items of the primary endpoint, Schirmer’s test, unstimulated salivary flow, ESSDAI, ESSPRI	At W28, 60% of pts achieved the primary endpoint. Mean ESSPRI score decreased from 6.4 (1.1) to 5.6 (2.0) (*p* = 0.0174). Mean ESSDAI score decreased from 8.8 (7.4) to 6.3 (6.6) (*p* = 0.0015) No differences in Schirmer’s test or unstimulated salivary flow	Prednisone (up to 10 mg/day), non-steroidal anti-inflammatory drugs, immunosuppressive/immunomodulatory or antimalarial agents, secretagogue therapy or tear substitutes were allowed	Minimum follow-up at 24 weeks; possible extension to 52 weeks
de Vita, 2015 [14]	19 pts	Open-label, multicenter phase II study	See Mariette, 2013	See Mariette, 2013		See Mariette, 2013	Thirteen of the 15 responders at W28 also responded at W52 (86.7%). At W52 improvement of 30% in the VAS dryness score remained unchanged Improvement in ESSDAI was maintained at 52 weeks	See Mariette, 2013	52 weeks
Mariette, 2022 [15]	86 pts	Phase II	four arms: placebo (13 pts), belimumab (24 pts), RTX (25 pts) and belimumab + RTX (24 pts)	> 18 year of age, diagnosis of SS (ACR/EULAR criteria), active disease (ESSDAI > 5)	13 pts	Primary: safety to week 68 Secondary: mean ESSDAI total score over time to week 68, the proportion of ESSDAI responders to week 68, the proportion of ClinESSDAI responders to week 68	Mean ESSDAI was lower with belimumab + rituximab than either other treatment groups. Change from baseline in ESSDAI total score was greater in the belimumab + rituximab group compared with belimumab and rituximab at every observation. Greater proportion of responders in the belimumab + RTX group than the rituximab group	Steroids	68 weeks
Belbezier, 2024 [27]	83 pts, 64 with non-systemic disease, 19 with systemic manifestations; 1 patient in the non-systemic disease group and 2 patients in the systemic group treated with belimumab	Retrospective		Patients with SS according to the ACR/EULAR criteria		Rate of development of systemic manifestations in the non-systemic SS group; improvement in ESSDAI in the systemic group	No development of systemic manifestation in the one case in the non-systemic group; significant improvement in both cases treated with belimumab the systemic group	Not clear	
Studies focusing on ANCA associated vasculitis									
Beketova, 2016 [17]	1pt with AAV	case report					Improvement of lung granulomatous lesions, no complete resolution at 12 months	Previous failure to CYC and RTX alone, intolerance to AZA and MMF	18 months
Jayne, 2019 [16]	106 pts with AAV	Multicenter, double blind, placebo-controlled	Patients on AZA 2 mg/kg and steroid < 10 mg randomized to either receive belimumab 10 mg/kg ev monthly (54 pts) or placebo (52 pts)	> 18 aa, diagnosis of GPA according to 2012 chapel Hill consensus, positivity for PR3 or MPO; previous induction with RTX or CYC		Primary: time to BVAS > 6, presence of ≥ 1 predefined major item on the BVAS Secondary: time to major relapse (one major item on BVAS), proportion of patients on remission at week 48	No difference in primary or secondary outcomes	GC < 10 mg, azathioprine	48 weeks or until major relapse
Studies focusing on cryoglobulinemic vasculitis									
de Vita, 2014 [19]	One patient with refractory SS, parotid low-grade B-cell MALT lymphoma and cryoglobulinemic vasculitis	Case report	RTX infusion after 49 infusions of belimumab alone				Complete resolution of parotid gland swelling and skin ulcers; improvement of polyneuropathy	Previous failure to rituximab alone, cyclophosphamide plus azathioprine, plasma exchange, rituximab plus corticosteroids, belimumab alone	3 years
Chevalier, 2020 [20]	3 pts with SS cryoglobulinemic vasculitis	Case series	Belimumab 200 mg/week after induction with rituximab 1 g	Diagnosis of SS complicated by cryoglobulinemic vasculitis, failure to at least one line of therapy			Patient 1: improvement of renal involvement, reduction in ESSDAI from 16 to 9. Patient 2: regression of cutaneous lesions, no worsening of polyneuropathia, ESSDAI decreased from 31 to 3. Patient 3: ESSDAI decreased (43/3)	Patient 1: rituximab 1 g; previous treatment with rituximab alone, cyclophosphamide and steroids. Patient 2: previous treatment with CYC, MMF, steroids, RTX. Induction with obinutuzumab, after 10 days belimumab. Patient 3: previous failure to rituximab alone, then CYC + RTX and after 14 days belimumab	Not specified
Saadoun, 2021 [21]	4 pts with refractory cryoglobulinemia vasculitis	Case series	Belimumab e.v. (10 mg/kg on days 0, 14, 28 and then every month) as add-on therapy, after failure of monotherapy with RTX	Serum cryoglobulinemia associated with the triad of purpura-arthralgia-asthenia Refractory cryoglobulinaemic vasculitis s defined as lack of symptoms improvement within 4–6 weeks after the initial treatment, or improvement < 50% after 12 weeks, presence of chronic manifestations after > 12 weeks of treatment			Patient 1: improvement in purpura and polineuropathy, normalization of C4 Patient 2: previous failure to rituximab alone (persistent arthralgias, persistent purpura); resolution of arhtralgias after 10 infusions (some transient purpuric episodes Patient 3: complete resolution of skin ulcers and purpura Case 4: complete resolution of purpura and arthralgias	Rituximab, GC (not specified the dosage)	
Izuka, 2021 [22]	1 pt with SS associated criogloblulinemic vasculitis	Case report	Belimumab 200 mg/week as maintenance (induction with RTX)				Resolution of purpura, arthralgia, proteinuria and hematuria	Previous RTX (suspended), HCQ 200 mg	6 months
Pouchelon, 2022 [18]	26 pts plus 7 patients extracted from review of the literature	Retrospective, multicenter study	11 patients received belimumab as salvage therapy (9 pts ev, 2 pts s.c.; 7 pts in combination with RTX, 4 pts as monotherapy	age ≥ 18 years at diagnosis of vasculitis; detectable type 2 or 3 mixed cryoglobulinemia at least on two assessments; histologically proven vasculitis, except in case of vascular purpura; disease refractory to RTX in combination or not with GCs		Complete clinical response: improvement of all clinical manifestations active before treatment Partial clinical response: improvement of at least 50% of baseline clinical manifestations	RTX + belimumab group: complete response in 6 out of 7 patients (86%); partial response in 1 out of 7 pts (14%) Belimumab group: complete response in 2 out of 4 patients (50%); failure in 2 out of 4 patients (50%)	Glucocorticoids: RTX + belimumab group:: median dose 20 mg Belimumab group: 4 mg (0–9)	Median follow-up after RTX failure was 26 months (IQR, 8–38 months) for the original cases and 24 months (IQR, 11–101) for the cases from the literature review
Studies focusing on Systemic Sclerosis									
Gordon, 2019 [23]	20 patients with dcSSc	Single-center, double-blind, placebo-controlled, pilot study	20 patients with dcSSc recently started on MMF randomized 1:1 to receive belimumab at 10 mg/kg intravenously or placebo; 9 patients in both groups completed follow-up	ACR criteria for SSc and 2013 ACR/EULAR for SSc; Age > 18 year old; disease duration of < 3 years baseline MRSS of ≥ 16		Primary: number of AEs and SAEs; difference in the median change in MRSS after 52 weeks of treatment Secondary: change in MRSS at 6 months; changes at 52 weeks in FVC and DLco and change at 52 weeks in patients’reported outcomes	Primary: no statistically significant difference in MRSS changes (In the belimumab group, 7 of 9 patients were clinical improvers compared to 3 of 9 patients in the placebo group (*p* = 0.153); no significant difference in the number of AEs or SAEs Secondary: no alterations of FVC or DLCO was recorded in both groups. Greater improvement in the SHAQ DI score	MMF	52 weeks
Mwangi, 2021 [24]	1 patient with SSc/LES overlap and CTD-ILD	Case report	Belimumab 750 mg e.v. monthly (add on to azathioprine)				Improvement in DLCO, TLC and 6MWT; improvement of ground glass opacities on chest HRTC	Previous treatment with cyclophosphamide, AZA	2 years
Studies focusing on inflammatory myositis									
Marder, 2024 [26]	17 pts with PM/DM	Multicenter randomized, double blind, placebo controlled clinical trial	Patients on standard of care (SoC) therapy were randomized 1:1 to IV belimumab 10 mg/kg (10 pts) or placebo (7 pts) for 40 weeks followed by 24 weeks belimumab 10 mg/kg in the open label phase	Age > 18 year old; definite or probable diagnosis of DM or PM (Peter and Bohan criteria); positivity for at least one typical auto-antibody. Refractory IIM was defined as inadequate response/intolerance to 3 months of glucocorticoids and/or at least one immunosuppressive agent. Standard Core Set Measures (CSM) with MMT8 < 125/150 were used to define active disease	7 pts	Primary endpoints: proportions of patients reaching a DOI and TIS at week 40 in belimumab arm vs. SoC alone arm Secondary endpoints: changes in DOI and/or TIS at week 64; prednisone dose change; safety profiles at 40 and 64 weeks	The proportion of patients reaching DOI by week 40 was numerically higher in belimumab arm (belimumab 33.3%/SoC 16.7%). Of patients in the belimumab group, 55.5% (5/9 patients) attained TIS 40 at week 40 and 44.7% (4/9 patients) reached TIS 40 at week 64. One-third of patients [33.3% (2/6 patients)] in the placebo arm attained moderate or major improvement by TIS at week 40, but no patients had any further alteration in their TIS at week 64 after crossing over to open-label belimumab at week 40 There was no steroid-sparing effect. No new safety signals were detected	Standard of care treatment (prednisone, MTX, MMF, AZA, IvIg)	40 weeks, 24 week open label extension
Yu, 2024 [25]	6 DM and 7 JDM pts refractory to conventional treatment	Retrospective	belimumab e.v. 10 mg/kg monthly	Bohan and Peter’s criteria, failure to at least one line of treatment	No	Reduction in Myositis Intention-toTreat Activity > 20% (primary) at week 12; reduction in steroid dose (secondary); improvement in cutaneous symptoms (secondary); reduction in CK (secondary)	Primary: 6 adult patients with DM and 6 out of the 7 patients with JDM (85.7%) achieved the primary outcome and remained clinically stable at week 24; Secondary: significant reduction in steroid use (DM: reduction by 78% at 24 weeks; Significant improvement in skin manifestation. Reduction on CK at 1 month	SoC	Minimum 24 weeks of follow-up (median follow-up 52 months)

*AAV* ANCA-associated vasculitis

*AZA* azathioprine

*BVAS* Birmingham Vasculitis Activity Index

*CTD*-*ILD* connective tissue disease-interstitial lung disease

*CYC* cyclophosphamide

*DLCO* diffusing capacity for carbon monoxide

*DM* dermatomyositis

*DOI* definition of improvement

*ESSDAI* EULAR Sjogren’s Syndrome Disease Activity Index

*FVC* forced vital capacity, *HCQ* hydroxicloroquine

*JDM* juvenile dermatomyositis

*MMF* mycophenolate mophetil

*mRSS* modified Rodnan Skin Score

*PM* polymyositis

*RTX* rituximab

*sAE* severe adverse event

*SoC* standard of care

*SOC* standard of care

*SS* Sjogren’s syndrome

*SSc* systemic sclerosis

*TIS* total improvement score

*TLC* total lung capacity

#### ANCA-associated vasculitis

Jayne et al. [16] conducted the only multicenter, double-blind, placebo-controlled study RCT evaluating belimumab as maintenance therapy in ANCA-associated vasculitis (AAV) associated with azathioprine on 105 subjects. Patients had already received an effective induction treatment with either rituximab or cyclophosphamide plus glucocorticoids and were randomized to either receive azathioprine (52 patients) or azathioprine plus belimumab (53 patients) as maintenance therapy. There was no significant difference in the rate of remission or the rate of vasculitis relapse in both groups.

A single case report [17] describes the use of belimumab for AAV in a patient with previous failure to cyclophosphamide and rituximab alone who was treated with belimumab in monotherapy; the author reported an improvement in the patient’s lung granulomatous lesions, yet without complete resolution. Thus, RTX was restarted, providing further improvement in CT images.

#### Cryoglobulinemic vasculitis

We retrieved a single retrospective study with a larger number of patients. The rest of the data was obtained by case series and case reports.

The most significant information on this topic comes from the study conducted by Pouchelon et al. [18]. The other data sources are case series and case reports [19–22]. Summarizing the available data, only four patients received belimumab as monotherapy, with a significant clinical response in only two of them. The response was assessed based on the resolution of vasculitis-related manifestations, particularly purpura and arthralgias since there are no validated disease activity index validated for clinical studies. In total, 16 patients received belimumab in combination with rituximab as induction therapy. Fifteen patients received belimumab/rituximab after rituximab alone, generally because of rituximab failure. One patient was administered belimumab for a total of 49 infusion and then was given rituximab. In all patients, there was a significant clinical improvement (either complete or partial response); see Table 2 for further details.

#### Systemic sclerosis

Gordon et al. [23] included 20 patients with early (disease duration < 3 years) diffuse cutaneous SSc (dcSSc) who had recently started treatment with mycophenolate mophetil. Patients were randomized either to receive belimumab or placebo. At 52 weeks, no significant reduction in the modified Rodnan Skin Score (mRSS) was observed between the belimumab and the placebo group.

One case report [24] described the use of belimumab in a patient diagnosed with SSc/SLE overlap with interstitial lung disease. The patient had previously failed several lines of treatment and a combination of belimumab plus azathioprine was introduced. After 2 years, there was a significant improvement in DLCO, TLC, 6MWT, and in the ground glass opacities on chest high resolution computer tomography (HRTC).

#### Inflammatory myositis

Yu et al. [25] retrospectively described 13 patients (6 pts with dermatomyositis, or DM, and 7 pts with juvenile dermatomyositis, or JDM) who were given belimumab as rescue therapy after failure of one or more lines of conventional or biological disease-modifying rheumatic drugs (cDMARDs or bDMARDs). All 6 adult patients with DM and 6 out of the 7 patients with JDM (85.7%) achieved the primary outcome (decrease in Myositis Intention-to-Treat Activity Index). A consistent improvement in cutaneous symptoms was observed in 5 out of 6 patients with DM (83.33%) and all 7 JDM patients (100%).

Marder et al. [26] conducted a randomized, double blind, placebo-controlled clinical trial, including 17 patients (13 polimyositis, 4 DM) who were randomized either to receive belimumab or placebo for 40 weeks, followed by 24 weeks of belimumab during the open label phase. Patients were already receiving standard of care therapy and had to be on a stable prednisone dose. Primary endpoints were the proportions of patients reaching a Definition of Improvement (DOI) and Total Improvement Score (TIS). These are composite scores based on muscle strength, muscle enzymes levels, patient’s and physician’s reported outcomes and evaluation of extra-muscular involvement.

Despite more patients within the treated arm reached the endpoint compared to controls, the result was not statistically significant (*p* = 0.60 for DOI and *p* = 0.99 for TIS).

#### Primary Sjogren’s syndrome

The BELISS trial was a multicenter phase 2 trial which included pSS patients with at least one the following criteria: systemic complications, early disease (≤ 5 years from symptom onset), or evidence of B cell activation. Twenty-eight patients received belimumab for 24 weeks, with continuation to week 48 in responders. A final follow-up visit was conducted at 52 weeks. Mariette et al. [13] reported the results at week 24 (28 subjects), whereas De Vita et al. [14] reported the results with follow-up at 52 weeks (19 subjects). Primary endpoints are reported in Table 1. At week 28, most subjects (18 patients, 60%) met the primary endpoint. ESSDAI scores significantly decreased (− 2.5; *p* = 0.0015), and even more so after excluding one patient who developed systemic sclerosis during follow-up (− 3.0; *p* < 0.0001). At 52 weeks, among the 15 responders at week 28, 87% maintained response. Improvements in ESSDAI and patient-reported outcomes (ESSPRI, SF-36 physical health) remained unchanged or slightly improved. B cell biomarkers remained stable, with a further decline in serum RF. Three out of four initial non-responders achieved response by week 52.

Belbezier et al. [27] retrospectively analyzed a cohort of 83 pSS patients, 64 without systemic manifestations (dry syndrome-arthromyalgia-asthenia’ triad alone) at baseline and 19 patients with extra-glandular manifestations at baseline. The subjects were treated with immunosuppressive therapy. Regarding non-systemic pSS subjects (64 pts, 24 untreated and 36 treated, of which one with belimumab) a significantly higher proportion of untreated patients, compared to treated subjects, developed systemic symptoms. The only patient treated with belimumab did not develop systemic symptoms during the 7-month follow-up. In this study, among the 19 systemic pSS patients, 2 were treated with belimumab. There was an overall clinical improvement in both patients.

Mariette et al. [15] studied the effect of the combination of rituximab and belimumab in patients with SS in a randomized, double-blind, phase 2, placebo-controlled 52-week trial. Patients were randomized in four arms (belimumab, rituximab, belimumab + rituximab, and placebo; see Table 2). There was a trend towards greater reduction in the ESSDAI score in all three arms, without any statistically significant difference. However, the ESSDAI score was lower in subjects treated with belimumab + rituximab at the end of the follow-up period (week 68), compared to either belimumab rituximab or placebo.

### Discussion

Belimumab currently represents a therapeutical option in patients affected by LES. Our SLR has highlighted the potential use of belimumab in different rheumatic diseases other than SLE, although the evidence is weak.

Belimumab may have therapeutic potential in rheumatic diseases characterized by aberrant B cell activation. The most convincing evidence available pertains to cryoglobulinemic vasculitis, despite the absence of RCT. B cell depleting therapies have become the first line treatments for cryoglobulinemic vasculitis [18, 28]. Understanding the role of belimumab could be interesting, considering its safety compared with B cell depleting agents such as rituximab. The only study addressing its use as monotherapy considered just 4 patients, with an efficacy rate of 50%, but we lack data from more robust trials. In most studies, belimumab was used after rituximab as maintenance therapy. In all cases, there was a significant clinical improvement, even when rituximab was not repeated after induction. This might offer a new alternative regimen especially for more frail patients.

Even in pSS patients, the BELISS-2 trial has demonstrated a significant reduction in ESSDAI both at 28 and 56 weeks. The consequent inclusion of belimumab in the 2020 EULAR recommendations is a significant step forward, however the lack of data from phase 3 trials warrants cautious interpretation [29]. In the other phase 2 trial available [15], although it was not the main purpose, in patients treated with belimumab alone there was a trend toward greater reduction in ESSDAI score compared with placebo.

Regarding inflammatory myositis, both studies from Yu et al. [25] and Marder et al. [26] have demonstrated some efficacy of belimumab in the management of these diseases. The trial conducted by Marder et al. failed to demonstrate statistically significance, but there was a small sample size and a higher-than-expected placebo response. In the other rheumatic conditions, such as SSc and AAV, belimumab failed to demonstrate any efficacy. However, several trials are being conducted at present on these diseases (Table 3).

**Table 3:** Ongoing clinical trial on the use of Belimumab

ID	Name of the study	State of the study
NCT03844061	Belimumab and Rituximab Combination Therapy for the Treatment of Diffuse Cutaneous Systemic Sclerosis	Recruiting
NCT05878717	A Study of the Efficacy and Safety of Belimumab in Adults with Systemic Sclerosis Associated Interstitial Lung Disease (BLISSc-ILD)	Recruiting
NCT06716606	A Study to Investigate the Long-term Safety and Efficacy of Belimumab in Adults with Systemic Sclerosis Associated Interstitial Lung Disease	Recruiting
NCT03967925	Rituximab and Belimumab Combination Therapy in PR3 Vasculitis (COMBIVAS)	Unknown status

One interesting possibility is to combine belimumab and rituximab treatment; it is unclear if dual therapy provides superior disease control compared to monotherapy in pSS and cryoglobulinemic vasculitis. Immunobiological evidence support a sequential scheme of administration with belimumab preceding rituximab, aiming to mainly target the microenvironmental BAFF in order to improve the success of the subsequent depleting treatment in the MALT pathologic tissue with rituximab. In a real pSS case, the sequential therapy with belimumab alone followed by rituximab alone led to a successful long-term clinical remission of lymphoma and cryoglobulinemic vasculitis, together with the persistent normalization of B cell hyperactivity and the disappearance of salivary gland swelling and cryoglobulinemia, both predictors of lymphoma in pSS [19].

The lack of conclusions regarding the safety and efficacy of belimumab in rheumatic diseases other than SLE is a consequence of data derived from small studies, often with heterogeneous methodologies and suboptimal study designs. This limitation is particularly evident in works evaluating cryoglobulinemic vasculitis and Sjögren’s syndrome, where the absence of phase 3 studies prevents definitive conclusions.

Additionally, the patient populations studied often exhibit marked clinical heterogeneity, forcing investigators to use composite indexes which are not always useful for clinical practice, in association to a wide variety of treatment regimens carried out before initiating belimumab therapy. This complexity makes it challenging to determine whether observed effects are attributable exclusively to belimumab.

Future research should focus on large, randomized phase 3 trials with an extended follow-up period to better define the role of belimumab in rheumatic diseases. Additionally long-term safety assessments are crucial to evaluate its impact on infection risk, immunomodulatory effects, and potential adverse events.

## Supplementary information

Below are the links to the electronic supplementary materials.

Supplementary file 1 (ODT 12.6 KB)

Supplementary file 2 (DOCX 269 KB)
