# Supplementary material for: Beyond systemic lupus erythematosus: a systematic review on the use of belimumab in other connective tissue diseases and vasculitides
Source: Clin Rheumatol. 2025 Dec 4;45(1):29–40. doi: 10.1007/s10067-025-07847-5 (PMC12855303; doi:10.1007/s10067-025-07847-5)
Supplement: Supplementary file 3 [file 10067_2025_7847_MOESM1_ESM.odt]

SUPPLEMENTARY MATERIALS

Supplementary table 1.

Patients exposure outcome (PEO) framework for eligibility of research question.

The PEO question format is used for qualitative research questions. Questions based on this format identify three concepts: [(1) Population, (2) Exposure, and (3) Outcome(s)](http://my.ucs.ac.uk/Library/Subject-Guides/Nursing,-Midwifery--ODP/PICO-Searching2.pdf).

Questions are outlined below:

- In SS patients (P), the use of belimumab (E) descreases ESSDAI (O)?

- In cryoglobulinemic vasculitis patients (P), the use of belimumab (E) decreases disease activity (O)?

- In SSc patients (P), the use of Belimumab (E), improves RSS (O)? 

- In PM/DM patients (P), the use of belimumab (E) decreases disease activity (O)?

- In AAV patients (P), the use of belimumab (E) increases the rate of remission (O)?

- In rheumatological patients other than SLE (P), the use of belimumab (E) increases the risk of adverse events, especially infections (O)?

Legend: SSc: Systemic sclerosis, RSS: Rodnan Skin Score; PM: polymyositis; DM: dermatomyositis; AAV: ANCA-associated vasculitis

Supplementary table 2.

Quality assessment of diagnostic accuracy studies (QUADAS) for articles included in the systematic review.

|  | bias patient selection | bias index test | bias reference standard | bias flow & timing | applicability patient selection | applicability index test | applicability reference standard |
| --- | --- | --- | --- | --- | --- | --- | --- |
| Yu, 2024 | 2 | 2 | 2 | 0 | 0 | 0 | 0 |
| Izuka, 2021 | 2 | 1 | 1 | 2 | 2 | 2 | 2 |
| Chevalier, 2020 | 2 | 1 | 1 | 2 | 2 | 2 | 2 |
| Jayne, 2019 | 0 | 0 | 0 | 0 | 0 | 0 | 0 |
| Mariette, 2022 | 0 | 0 | 0 | 0 | 0 | 0 | 0 |
| Beketova, 2016 | 1 | 1 | 0 | 0 | 2 | 2 | 2 |
| Belbezier, 2022 | 0 | 0 | 0 | 0 | 2 | 0 | 0 |
| Marder, 2024 | 0 | 0 | 0 | 0 | 0 | 0 | 0 |
| De Vita, 2014 | 0 | 0 | 0 | 2 | 2 | 2 | 0 |
| Mwangi, 2021 | 0 | 2 | 2 | 2 | 2 | 2 | 2 |
| Gordon, 2019 | 0 | 0 | 0 | 0 | 0 | 0 | 0 |
| Saadoun, 2021 | 0 | 2 | 0 | 2 | 2 | 2 | 0 |
| De Vita, 2015 | 0 | 0 | 0 | 0 | 0 | 0 | 0 |
| Mariette, 2013 | 0 | 0 | 0 | 0 | 0 | 0 | 0 |
| Pouchelon, 2022 | 0 | 0 | 0 | 2 | 0 | 0 | 0 |

QUADAS-2 comprises 4 domains: patient selection, index test, reference standard, and flow and timing. Each domain is assessed in terms of risk of bias; and the patient selection, index test, and reference standard domains are also assessed regarding applicability. O= low risk; 1= high risk; 2= unclear risk.
